# Predictive Neuromarker Patterns for Calcification Metaplasia in Early Tendon Healing

**DOI:** 10.3390/vetsci11090441

**Published:** 2024-09-19

**Authors:** Melisa Faydaver, Valeria Festinese, Oriana Di Giacinto, Mohammad El Khatib, Marcello Raspa, Ferdinando Scavizzi, Fabrizio Bonaventura, Valentina Mastrorilli, Paolo Berardinelli, Barbara Barboni, Valentina Russo

**Affiliations:** 1 Unit of Basic and Applied Biosciences, Department of Biosciences, Agro-Food and Environmental Technologies, University of Teramo, 64100 Teramo, Italy; valeria.festinese@unicam.it (V.F.); odigiacinto@unite.it (O.D.G.); melkhatib@unite.it (M.E.K.); pberardinelli@unite.it (P.B.); bbarboni@unite.it (B.B.); vrusso@unite.it (V.R.); 2 National Research Council (CNR), Campus International Development (EMMA-INFRAFRONTIER-IMPC), Institute of Biochemistry and Cellular Biology (IBBC), 00015 Monterotondo Scalo, Italy; mraspa@emma.cnr.it (M.R.); fscavizzi@emma.cnr.it (F.S.); bonaventura@emma.cnr.it (F.B.); 3 Plaisant S.r.l., 00128 Rome, Italy; valentina.mastrorilli91@gmail.com

**Keywords:** Achilles tendon, mice, neuromarkers, animal models, metaplasia, chondrocyte-like cells, calcification

## Abstract

**Simple Summary:**

Tendon injuries lead to poor healing with scarring and occasionally calcification. In these metaplastic drifts, mice with injured Achilles tendons (ATs) represent a good preclinical model since about one-third of tissues show calcium deposits (i.e., non-calcified vs. calcified tendons) at 28 days post-injury (p.i.). No information has been collected to date about the role exerted in metaplasia by neuromarkers, which, on the contrary, modulate tendon healing in diverse animal models. In particular, it remains to be clarified whether specific patterns in non-calcified/calcified tendons could be predictive of the tissue healing fate. The present results show that neuromarkers such as neurofilament-200 (NF200), nerve growth factor (NGF), neuropeptide Y (NPY), galanin (GAL), and calcitonin gene-related protein (CGRP) had a dynamic (7 vs. 28 days p.i.) and differentiative expression during early tendon healing. In detail, in non-calcified tendons, NF200 values were similar to pre-injury, whereas, in calcified tendons, NF200 persisted at high levels. Notably, a high CGRP/NPY (vasodilatory/vasoconstrictive) neuromediator ratio characterised tendon healing towards calcification. A novel finding was the contribution of tendon cells in the synthesis of neuromarkers, suggesting a connection between the cells and the nervous system. This research offers novel insights into neurobiological mechanisms during early tendon healing in mice and identifies a neuromarker pattern predictive of tendon metaplastic repair.

**Abstract:**

Unsuccessful tendon healing leads to fibrosis and occasionally calcification. In these metaplastic drifts, the mouse AT preclinical injury model represents a robust experimental setting for studying tendon calcifications. Previously, calcium deposits were found in about 30% of tendons after 28 days post-injury. Although a neuromediated healing process has previously been documented, the expression patterns of NF200, NGF, NPY, GAL, and CGRP in mouse AT and their roles in metaplastic calcific repair remain to be explored. This study included a spatiotemporal analysis of these neuromarkers during the inflammatory phase (7 days p.i.) and the proliferative/early-remodelling phase (28 days p.i.). While the inflammatory phase is characterised by NF200 and CGRP upregulation, in the 28 days p.i., the non-calcified tendons (*n* = 16/24) showed overall NGF, NPY, GAL, and CGRP upregulation (compared to 7 days post-injury) and a return of NF200 expression to values similar to pre-injury. Presenting a different picture, in calcified tendons (*n* = 8), NF200 persisted at high levels, while NGF and NPY significantly increased, resulting in a higher NPY/CGRP ratio. Therefore, high levels of NF200 and imbalance between vasoconstrictive (NPY) and vasodilatory (CGRP) neuromarkers may be indicative of calcification. Tendon cells contributed to the synthesis of neuromarkers, suggesting that their neuro-autocrine/paracrine role is exerted by coordinating growth factors, cytokines, and neuropeptides. These findings offer insights into the neurobiological mechanisms of early tendon healing and identify new neuromarker profiles predictive of tendon healing outcomes.

## 1. Introduction

Tendinopathy is a disease that affects both human and animal patients [1,2], especially athletes, labourers, and older adults [3,4]. This pathology leads to significant performance issues and economic losses [5], impacting mobility and quality of life [6]. When it comes to injury recovery, conventional treatments, including both conservative and surgical methods, often have poor clinical outcomes due to a low tendon repair response, which frequently leads to fibrotic resolution [7,8] or occasionally to development of calcium deposits [9]. Indeed, recent studies have shown that the incidence of the calcification process varies at between 2.7% and 20% in human tendon tissues [10,11,12] and approximately 30% in the mouse Achilles tendon (AT) injury model [13]. The exact mechanism behind the formation of calcification foci in tendons is unknown, but it is suggested that inflammation and hypoxia are key factors in the development of heterotopic mineralisation [14,15].

Recently, it has been also found that the nervous system in tendons plays a collaborative role in not only regulating homeostasis but also modulating the healing process by regulating vascularisation, hypoxia, inflammation, and pain, influencing the tissue repair outcome [16,17,18,19,20,21,22,23]. The nervous system’s involvement extends beyond afferent (sensory) functions to include efferent pathways that interact with various neuromarkers. Indeed, in a system biology study on tendon development among different model organisms, the nervous system has recently been identified as crucial in orchestrating early tenogenesis, operating through either species-specific or conserved driver molecules [16,17]. The nervous system takes action when tendon tissue deviates from its homeostatic state and these molecules undergo a series of changes triggering physiological and pathological pathway cascades [18]. In the most extensively studied mammalian species, rat and humans, the levels of neuromodulators and neuropeptides undergo dynamic changes during tendon healing corresponding to different phases, namely the inflammatory, proliferative, and remodelling phases [18,19,20,21]. In detail, neuropeptides like nerve growth factor (NGF) and neurofilament-200 (NF200), and neuromediators, such as calcitonin gene-related peptide (CGRP), neuropeptide Y (NPY), and galanin (GAL), have been identified to play a pleiotropic role in orchestrating tendon healing [19]. During the healing process, these neuromarkers help modulate the early inflammatory response and long-term extracellular matrix (ECM) remodelling, contributing to the overall tendon repair. Indeed, the early phase of tendon healing is characterised by extensive nerve ingrowth into the tendon proper, leading to the time-dependent appearance of autonomic and glutamatergic mediators, which amplify and regulate inflammation and tendon regeneration [18,19]. If not appropriately modulated, this extensive nerve ingrowth and expression of different neuromarkers can lead to pro-inflammatory, nociceptive, degenerative, and metaplastic tissue responses [22,23]. The preliminary information collected to date, on the one hand, highlights the neurogenic role underlying tendon regeneration, but also emphasises how a better comprehension of neuromarker distribution and timing during tendon healing is essential for unravelling the neurogenic mechanisms, offering valuable insights into the identification of prognostic biomarkers as well as supporting the development of new potential therapeutic targets for tendon disorders [18].

To delve into the physiological and pathological profiles of neuromarker expression, it is essential to identify a preclinical experimental model that provides rigorous information about the success and failure states of the tendon healing process. In this regard, the mouse AT injury model plays a fundamental role, as it allows for the observation and comparison of tissues undergoing a healing process versus those with a compromised reparative process. In fact, in a recent study [13], a positive correlation between locomotor activity and key quantitative morphometric parameters was observed, shedding light on the processes of cell alignment and ECM remodelling during the early stage of the tendon healing process. In fact, this effect was particularly enhanced in animals with bilateral tendon injuries, likely a result of the distinct adaptive movement imposed by the bilateral mechanical tissue defects, and free access to movement. Of note, this study also highlighted that 30% of the analysed samples, independently of the injury type (unilateral or bilateral) or the movement availability, showed calcification processes [13]. This underscores the importance of the mouse Achilles tendon model for studying the distribution, timing and pattern of neuromarkers during tendon healing and in both non-calcified and calcified samples.

Based on these premises, since a comprehensive assessment of neuromarker expression had not yet been performed in mice, the focus of this research centred on elucidating the distribution and expression patterns of the main neurogenic markers, such as NF200, for nerve ingrowth, and NGF, CGRP, NPY, and GAL, to verify their involvement in the repair process of the described mouse AT model [13]. In particular, this study at first aimed to provide comprehensive insights into the spatiotemporal dynamics of these molecules across various stages of tendon healing: inflammatory (7 days p.i.) and proliferative/early remodelling (28 days p.i.) stages [13,19]. Furthermore, in the latter stage, we verified whether a differential modulation of the analysed neuromarkers occurred between non-calcified tissues and tendons with signs of calcification foci. The outcomes of this study address the existing knowledge gap concerning the mouse tendon model, contributing to a better understanding of neurogenic involvement in the mouse tendon repair process and identifying neuromarker patterns that might have prognostic value for tendon healing.

## 2. Materials and Methods

### 2.1. Experimental Plan

In this research, specific pathogen-free (SPF) CRL: CD1 (ICR) male mice around 10–12 weeks old were used. These mice were accommodated in the EMMA/Infrafrontier international research infrastructure situated at the CNR-IBBC Core Structure in Monterotondo, Rome, Italy. The use of SPF mice in this study ensured that observed changes in the tendon repair process were not influenced by microbial factors or pathogens. A more comprehensive description of the housing and husbandry practices can be found in a previously published article [13]. A total of 24 mice with bilateral Achilles-tendon-induced injury were used, resulting in the analysis of 48 tendons. In detail, two time points were considered for the analysis: 7-day-old (12 animals/24 tendons) and 28-day-old injury (12 animals/24 tendons). Additionally, 8 mice (equating to 16 healthy tendons) were included in the control group (CTRL). The total of analysed animals, including controls, was 32. The sample size was calculated using G*Power (version 3.1.9.7.) [24]. Tendon explants were performed after 7 and 28 days p.i., aimed at capturing the inflammatory and proliferative/early remodelling phases, respectively.

### 2.2. Surgical Procedure

**Figure 1:**
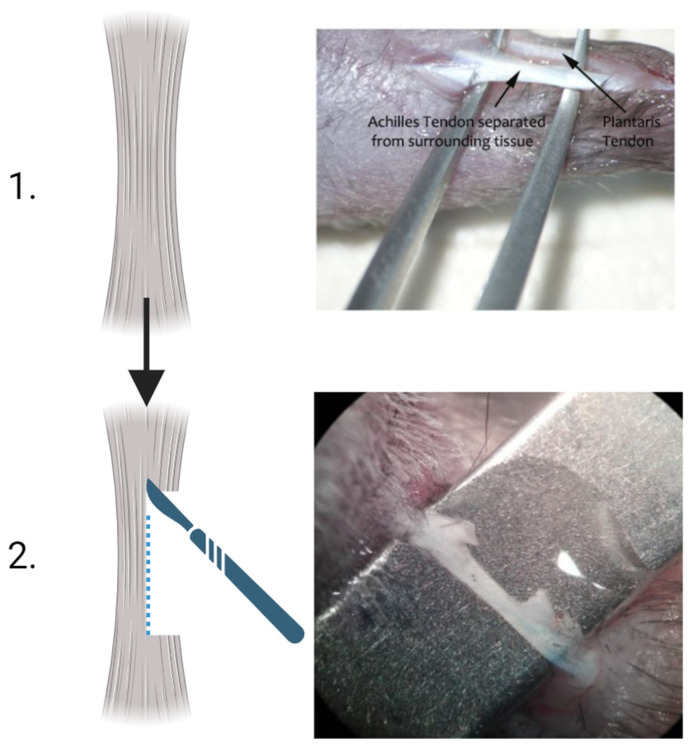
Illustrative images of the tendon injury in the mouse AT model. (**1.**) The tendon was identified, exposed, and isolated from adjacent tissues. (**2.**) A controlled lesion, not exceeding 50% of the total tendon diameter, was created at the mid-length of the tendon using a scalpel blade. The images were obtained using a stereomicroscope during the surgical procedure.

All surgical procedures were carried out using a validated protocol according to Faydaver et al. [13]. In detail, strict aseptic protocols were followed, and surgeries were conducted under general anaesthesia, with analgesia for both intraoperative and postoperative pain management. Anaesthesia was induced and maintained using a 4% isoflurane/oxygen mixture initially, followed by a 1.5–2% isoflurane/oxygen mixture during the procedure. Animals were placed in a sternal position on a heating pad, with hindlimbs extended caudally, under a stereomicroscope for optimal visualisation (Figure 1). Following trichotomy and skin disinfection, a 6/7 mm incision was made along the posterior aspect of the leg, between the musculotendinous junction of the gastrocnemius muscle and the calcaneus. The Achilles tendon (AT) was then carefully identified, exposed, and isolated from adjacent tissues (Figure 1). A controlled lesion, not exceeding 50% of the total tendon diameter, was created at the mid-length of the tendon using a scalpel blade. Throughout the procedure, the tendon was kept moist with a sterile saline solution. The incision was closed with 6–0 monofilament sutures. Postoperatively, mice were allowed immediate ambulation to facilitate tissue regeneration, while also administering buprenorphine at 0.05 mg/kg and an antibiotic (enrofloxacin at 0.2 mg/kg). Although the animals were of the SPF health category and thus pathogen-free, reducing their susceptibility to postoperative infections, these precautions were taken to ensure optimal recovery.

### 2.3. Histological Analysis of Explanted Tendon ECM

The entire ATs, including the myotendinous junction and the enthesis, were explanted and were carefully isolated from the surrounding tissues to ensure the integrity of the samples. Histological analysis was performed following previously validated protocols [13]. Briefly, ATs from healthy animals and all experimental groups were fixed in 4% paraformaldehyde/phosphate-buffered saline (PBS) for 1 h. Subsequently, they were embedded in paraffin wax using routine processing [13]. For histological analysis, samples were sectioned longitudinally to a thickness of 7 μm using a microtome and mounted on polylysine-coated microscope slides. The paraffin was removed by immersion in xylene for 10 min, followed by rehydration using a descending alcohol series (100% to 70%), and then washed with distilled water.

Histological staining techniques included the following:

- Haematoxylin–Eosin (H-E), for a standard histological observation [25].
- Alcian Blue 1% *w*/*v* solution in acetic acid pH 2.5, which stains acidic polysaccharides (especially glycosaminoglycans) and serves as an indirect marker for chondrocyte cells according to commercially available kit used (Bio-Optica S.p.A-20134, Milano, Italy) [26].
- Alizarin Red, used to detect the presence of calcium deposits in the tissue [27].

### 2.4. Immunofluorescence Assessment of Neurogenic Markers

The tissue sections obtained as described in the histological analysis section were used also for immunofluorescence (IF) studies.

IF studies were conducted using the antibodies described in Table 1 on mice tendon samples following the protocol mentioned below.

The IF staining included:

- Sensory innervation: Calcitonin gene-related peptide (CGRP);
- Opioid-like signalling: Galanin (GAL);
- Autonomic innervation: Neuropeptide Y (NPY—Sympathetic);
- Neuropeptides: Nerve growth factor (NGF), Neurofilament-200 (NF200);
- Mature tenocyte-related marker: Tenomodulin—for the double IF for intra-cellular positivity.

#### 2.4.1. Single Immunostaining

Following PBS washing, trypsin was used for antigen unmasking (1 mg/mL in PBS) at room temperature (RT) for 15 min. Non-specific binding was blocked by incubating the sections in PBS/1% bovine serum albumin (BSA) for 1 h. Subsequently, the tissue sections were incubated with the primary antibody overnight at RT, followed by exposure to the secondary antibody for 1 h at 4 °C. Nuclei were stained with DAPI (Sigma-Aldrich, St. Louis, MO, USA, D9542, 1:100) for 10′ at RT. Negative controls were obtained, by omitting the primary antibody but adding the secondary antibody. All negative controls proved to be negative.

#### 2.4.2. Double Immunostaining

The samples were prepared and incubated with the primary and secondary antibodies following the above-mentioned protocol. After the secondary antibody incubation period, the samples were fixed with 4% formaldehyde for 10′ at RT, and then washed and blocked again using PBS/BSA 1%, followed by incubation of the primary antibody overnight and the secondary antibody for 1h at RT. The use of two secondary antibodies was necessary for the double IF staining performed with tenomodulin and each neuromarker. This required the use of a green channel (Alexa Fluor 488) for the neuromarker’s antibody and a red channel (Cy3) for TNMD, allowing for the simultaneous visualisation of both markers. Nuclei were stained with DAPI (Sigma-Aldrich, St. Louis, MO, USA, D9542, 1:100) for 10′ at RT. Negative controls were obtained by omitting the primary antibody but adding the secondary antibody. All negative controls proved to be negative.

### 2.5. Fluorescence Quantification Analysis

**Figure 2:**
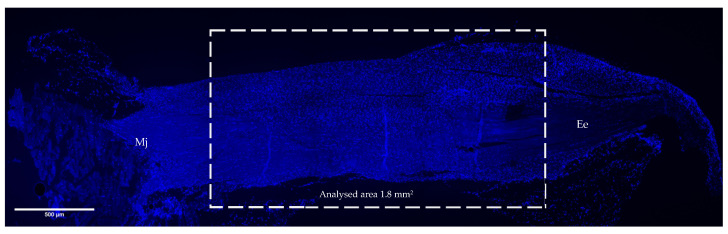
Example of a detailed large-format image representing an injured tendon tissue in high resolution. The quantification analysis was conducted in a defined area of 1.8 mm^2^ (length × width: 3 mm × 0.6 mm), and the myotendinous junction (Mj) and the enthesis (Ee) were not considered. Large images (LIs) were acquired using the Timelapse Nikon fluorescent microscope at 4× magnification, and then 12 detailed images within the considered area were taken of each tendon in the analysed area at 40× magnification. Scale bar = 500 µm.

To perform the fluorescence quantification analysis and gain a comprehensive image of the entire tendon, large high-resolution images were captured using the Timelapse Nikon fluorescent microscope at 4× magnification, which allowed the definition of the area to be analysed (large image: LI). The middle of the tendon representing the centre of the lesion was measured and analysed, while extremities of the myotendinous junction (Mj) and the enthesis (Ee) were not considered. The mean analysed area of each tendon was 1.8 mm^2^ (length × width: 3 mm × 0.6 mm) (Figure 2). Subsequently, detailed images were obtained from each tendon within the analysed area at 40× magnification for the quantification analysis. A minimum of 12 random images from each sample were taken, spanning all tested groups. The images were acquired using the same exposition, contrast, and light intensity for all analysed markers. Following previously validated protocols [13,28], the fluorescence intensities of the samples, subjected to immunostaining for NGF, NF200, GAL, CGRP and NPY, were evaluated using the RGB Profiler plugin within the ImageJ software (NIH). Each image captured underwent processing through this plugin, generating red, green and blue profile plots on a unified graph for each image. The results were expressed in average fluorescence intensity (aFI), providing a visual representation of both the minimum and maximum fluorescence values. Using the negative control of each sample, the minimum fluorescence intensity level was removed from the overall fluorescence expression results.

### 2.6. Statistical Analysis

Data analysis was conducted using GraphPad Prism 9 software (GraphPad Software, San Diego, CA, USA). A one-way ANOVA was performed on the fluorescence level values obtained from the samples. The results were graphically represented, and statistical significance was determined at a threshold of *p* < 0.05.

## 3. Results

### 3.1. Histological Assessment of the Tendon ECM

**Figure 3:**
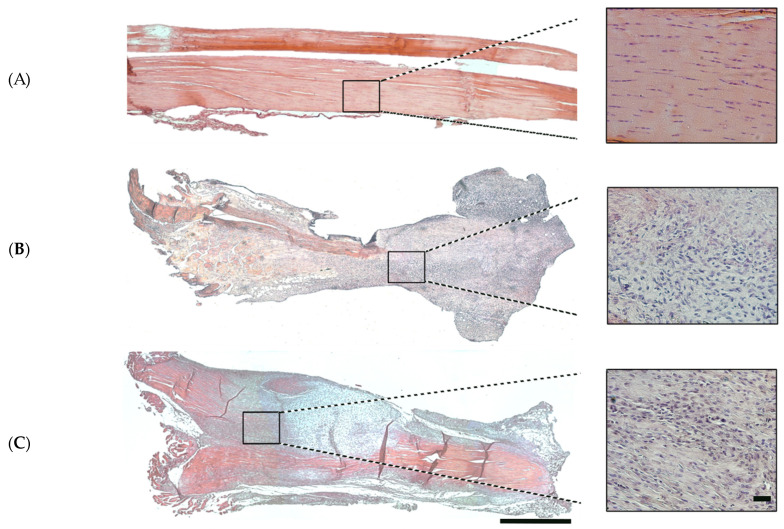
Representative large images (LIs) of the entire mouse ATs stained with H-E for histological analysis. (**A**) Healthy tendons and (**B**) samples after 7 days post-surgery and (**C**) 28 days post-surgery. The squares in each sample represent the position of the detailed image with magnification (right). The detailed images show the cellularity and fibre disposition in that area. LI scale bar = 500 µm. Magnified image scale bar = 50 µm.

The organisation of the ECM of the analysed samples, healthy and post tendon injury (7 and 28 days), was evaluated with H-E staining. The healthy tendons were characterised by a low cell density and an organised ECM (Figure 3). The tendon fibres appeared densely packed and aligned parallel to the longitudinal axis of the tendon (Figure 3A). In contrast, tendon samples at 7 days p.i. exhibited a different histological morphology indicative of the acute phase of injury. These changes included hypercellularity with the presence of more round cells and a completely disorganised ECM (Figure 3B). The 28-day p.i. phase marked a shift towards tissue repair and remodelling. During this stage, hypercellularity remained elevated; however, the ECM started to reorganise, signifying the initiation process of tissue repair (Figure 3C).

**Figure 4:**
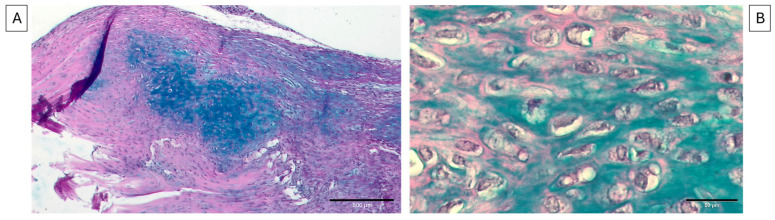
Representative images of Alcian Blue staining obtained from tendons at 28 days p.i. The staining in image (**A**) (scale bar = 500 μm—10×) shows the positivity to Alcian Blue in the tendon, and (**B**) shows a detailed 20× image of the chondrocyte-like cells within spontaneously healed tendons (scale bar = 50 μm). The positivity is visible as a bright blue staining surrounding the chondrocyte-like cells in the ECM, where proteoglycans are released.

Following the H-E results, a more in-depth analysis of the metaplasia process was performed, using Alcian Blue and Alizarin Red staining. Specifically, Alcian Blue staining showed that chondrocyte-like cells were absent from both healthy tendon samples and those collected at 7 days p.i. whereas cells positive for Alcian Blue staining were exclusively observed in all samples collected at 28 days p.i. (Figure 4A—10×), confirming our previous data [13]. In particular, these cells exhibited a characteristic rounded or oval shape with dark, purple-stained nuclei and cytoplasmic extensions into the surrounding ECM, as depicted in Figure 4B (20×). Alcian Blue staining highlighted the presence of proteoglycans secreted by these cells, indirectly indicating the presence of chondrocyte-like cells within the tendon tissue.

**Figure 5:**
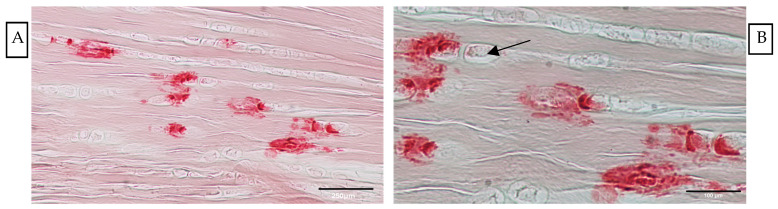
Representative images of Alizarin Red staining showing (**A**) calcium deposits within the tendon tissue at 28 days p.i. and surrounding the nuclei (arrow). Scale bar = 250 µm. (**B**) The presence of calcium deposits arranged around the chondrocyte-like cells. Scale bar = 100 µm.

The Alizarin Red staining highlighted, exclusively at 28 days p.i., the presence of calcium deposits in about 30% of healing tendons analysed (8 out of 24) (Figure 5). The foci of calcifications were observed within the tendon proper encircled by chondrocyte-like cells, manifesting as structural irregularities embedded within the cellular matrix (Figure 5).

### 3.2. Neuromarkers’ Distribution within the Healing Tendons during the Inflammatory and Proliferative/Early Remodelling Phases

**Figure 6:**
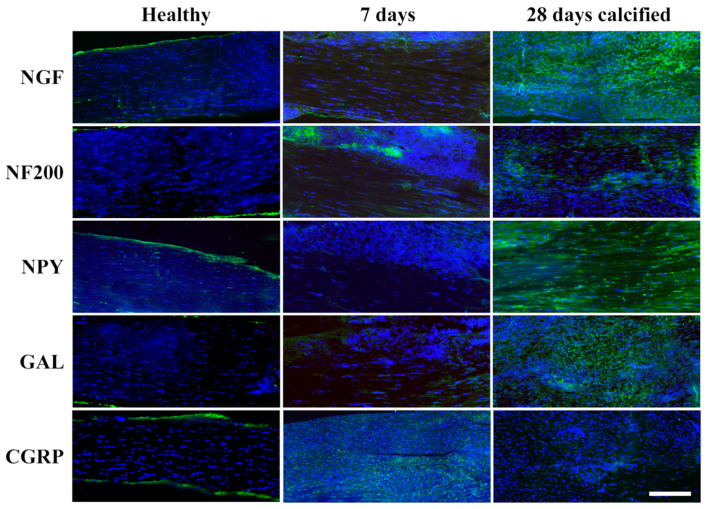
Exemplary LI images of samples showing the neuromarker distribution in healthy, 7-day and 28-day tendons. For the 28-day time point, only calcified samples are shown as an example. In the healthy tendon (left), positivity can be observed concentrated in the paratenon and endotenon, whereas, in the rest of the images, fluorescence expression can be observed in the tendon proper. Scale bar = 200 µm. Green channel (AlexaFluor 488) represents the each analysed neuromarker, whereas the nuclei are stained with DAPI (blue).

The expression of neuromarkers (NGF, NF200, GAL, CGRP, NPY) was examined in healthy, 7 days p.i. and 28 days p.i. non-calcified (*n* = 16) and calcified (*n* = 8) tendons. In the healthy tendons, the expression of the neuromarkers was observed in all samples, but the positivity was mainly located within the paratenon (Figure 6 Healthy). In contrast, a diffuse expression was evident for all the analysed neuromarkers at both 7 and 28 days p.i., independently of the presence of calcification (Figure 6). In particular, the expression was detected in the tendon proper and the ECM. Furthermore, neuromarker positivity was also detected in the cytoplasm of some of the cells belonging to tendon tissue. Notably, the intra-cellular expression was not observed in the healthy tendon but was identified in both samples at 7 days p.i. and in calcified and non-calcified samples at 28 days p.i. In order to verify any co-localisation of the neuromarkers with tenocytes, TNMD, a tenocyte-related marker, was used in a dual IF analysis.

**Figure 7:**
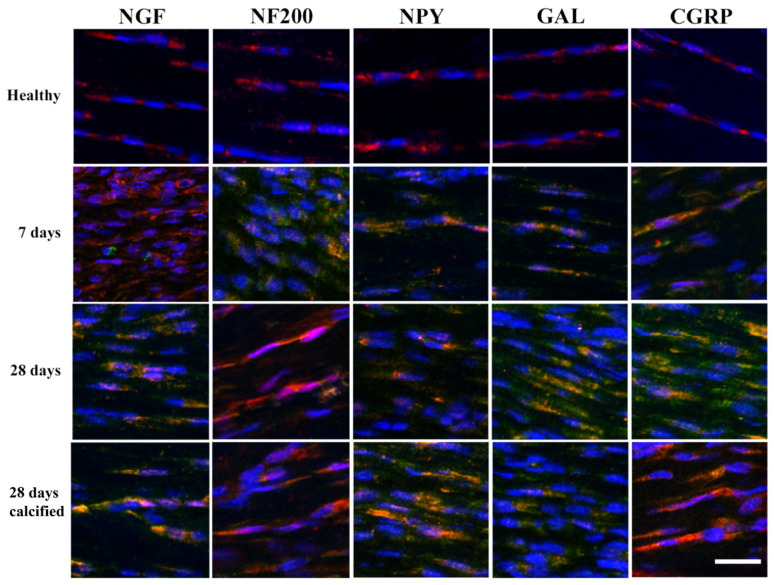
Neuromarker double IF co-staining TNMD in healthy tendons and the various stages of tendon healing (7 days, 28 days non-calcified and 28 days calcified samples). Tenocyte positivity using double staining with TNMD (red channel—CY3) together with the individual neuromarkers’ staining (green channel—AlexaFluor 488) can be observed. When the markers proved to be co-localised, orange fluorescence was evident. Scale bar = 25 µm. The green channel (Alexa Fluor 488) represents each analysed neuromarker, and the red channel (Cy3) was used for TNMD, whereas the nuclei were stained with DAPI (blue fluorescence).

The analysis carried out on healthy tendons showed a lack of merged fluorescence between TNMD and neuromarkers, indicating no neurogenic contribution by tenocytes. On the contrary, tenocytes (TNMD-positive cells) displayed a co-expression of neuromarkers in all tendon explants both at 7 and 28 days p.i. (Figure 7).

**Figure 8:**
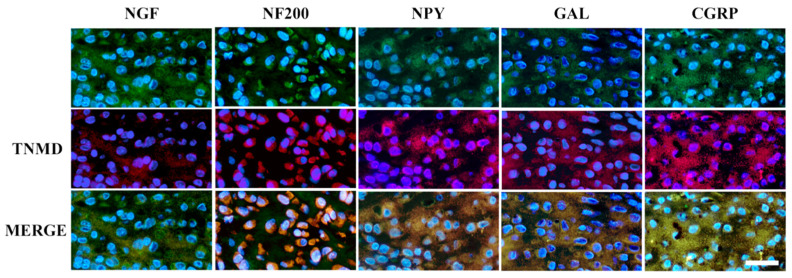
Double IF staining of each analysed neuromarker together with TNMD in chondrocyte-like cells. These images show a high positivity level in chondrocyte-like cells for both TNMD and each neuromarker. Moreover, there is a high intra-cellular positivity, as well as ECM positivity. Scale bar = 50 µm. The green channel (AlexaFluor 488) represents each analysed neuromarker, and red (cy3) is for TNMD, whereas the nuclei are stained with DAPI (blue).

Moreover, TNMD positivity was also observed in chondrocyte-like cells, as well as intra-cytoplasmatic neuromarker co-localisation at 28 days p.i. (Figure 8).

While other neuromarkers expressed a more diffuse pattern in the ECM, NF200 displayed a distinctive positivity revealing the presence of nerve fibres and nerve bundles, especially at 28 days p.i.

### 3.3. Neuromarkers’ Expression during the Inflammatory, Proliferative/Early Remodelling Stage of Spontaneous Healing

**Figure 9:**
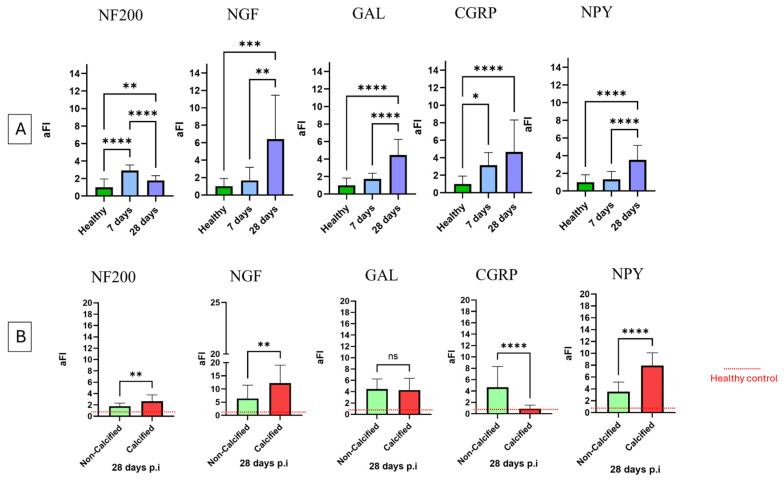
Graphical representation of the average fluorescence intensity (aFI) of each analysed sample. (**A**) aFI from healthy vs. 7 days vs. 28 days non-calcified p.i.; (**B**) 28 days p.i. non-calcified vs. calcified tissues normalised to the healthy tendon sample. In the statistical analysis, significance was expressed in * (*p* < 0.05), ** (*p* < 0.01), *** (*p* < 0.001), **** (*p* < 0.0001). Each sample was compared with the others.

The quantification of aFI was carried out in healthy vs. 7 days (inflammatory phase) and 28 days p.i. tendons (proliferative/early remodelling stage) (Figure 9A). The explants isolated at 28 days p.i. were also classified into the two previously distinct categories based on the presence or absence of calcified foci (Figure 9A). The analysis of aFI revealed two diverse modalities of expression of neuromediators: NF200 and CGRP showed an early response characterising the inflammatory phase of tendon healing, while NGF, NPY and GAL were mainly present during the proliferative/early remodelling stage (Figure 9A). In addition, neurogenesis was strictly dependent on the fate of tissue remodelling, with all neuromarkers showing differential expression in samples with or without calcification, except for GAL (Figure 9B). In detail, as shown in Figure 9A, the aFI of NF200 increased at 7 days p.i. (7 days vs. healthy *p* < 0.0001) only to then return to the basal value in tendons approaching early remodelling without any signs of calcification *(p* < 0.01). On the contrary, high aFI levels persisted at 28 days in calcified tendons (28 days vs. healthy *p* < 0.001 and 28 days vs. 7 days *p* < 0.0001) (Figure 9B). CGRP displayed an early rise in expression (7 days vs. healthy *p* < 0.01) (Figure 9A) only to then further increase exclusively in non-calcified explants (Figure 9B) while significantly decreasing in calcified ones (*p* < 0.001), reaching levels of aFI pre-injury (vs. healthy *p* > 0.05). NGF, NPY and GAL showed no early increase (for all 7-day vs. healthy *p* < 0.05), while a clear increase in aFI was observed at 28 days p.i. (for all 28 days vs. healthy or vs. 7 days at least *p* < 0.05). Interestingly, the comparison of NGF and NPY aFI between non-calcified and calcified tissues at 28 days revealed a differential expression p.i. (Figure 9B). Specifically, a significant increase in both was recorded in tissues developing calcified foci (Figure 9B). Differently, aFI GAL (Figure 9B) did not show any differences between the two 28-day groups, demonstrating that the expression of opioid-like signalling was independent at least during the first 28 days of healing from the calcification fate of the remodelling phase.

## 4. Discussion

The present research aimed to assess the spatiotemporal expression pattern of key neuromarkers on mice with experimentally injured ATs. Differently from other species, the mouse preclinical tendon injury model has not yet been explored in a way specifically addressing different stages of spontaneous healing in tendons with diverse clinical outcomes. This highlights a significant gap in our understanding of the neural mechanisms leading to diverse tendon healing outcomes using an animal model of high translational value [13]. Specifically, this research clarifies the mechanisms that contribute to the key signalling pathways regulating early tendon healing [29]. Furthermore, the release of neurotransmitters and nerve growth factors can directly promote or inhibit cellular processes by impacting the success of the proliferative/early remodelling phase [19]. Thus, understanding how these neuromarkers influence successful or unsuccessful (calcification) tendon healing can provide novel evidence when identifying predictive prognostic markers of the tendon reparative process. In this context, the mice AT injury model offers the unique opportunity of studying the mechanism involved in calcification as drift of tendon healing, since a third of bilaterally injured tendons presented this type of metaplasia at 28 days p.i. [13].

The value of this study lies in defining the spatiotemporal expression patterns of key neuromarkers during spontaneous mice tendon healing. The first goal of this research was to focus on the expression levels of NF200, NGF, CGRP, GAL and NPY before and after experimentally induced injury, and their relation to tendon healing outcomes. Dynamic regulation of neurogenic elements was defined throughout the tendon healing process, specifically during the transition from the inflammatory (7 days p.i.) to the proliferative/early remodelling phases (28 days) when the calcific drift may occur [13,19]. Indeed, spontaneous tendon healing began with an initial phase characterised by significant cellular infiltration and a disorganised ECM. It is also recognised that, in this phase, high levels of activated innate immune cells are present within the disorganised ECM [30]. Then, healing tendons exhibited initial signs of remodelling characterised by a progressive reduction in cellular presence, and the accumulation of a progressively more organised ECM around day 28 p.i., confirming a previous study on mice that also showed remodelling of the blood vessel network [13]. All samples exhibited chondrocyte-like cells during this latter AT healing time point, and in some of the tissues (8 out of 24 in the present research), areas of calcification, confirming the 30% incidence previously described in mice [13].

The neuromarkers were already present in healthy tendons, albeit at relatively low levels, but only specifically located in the paratenon and endotenon, confirming observations reported in other species [31,32,33,34]. At 7 days p.i., NF200 and CGRP were the earliest upregulated neuro-molecules. Increased levels of NF200 suggest a neurogenic response to tissue damage, whereas CGRP, known for its vasodilation role, stimulates the proliferation and migration of endothelial cells, thereby contributing to enhancing the blood supply immediately after injury [35]. The low levels of NPY recorded in this phase are also compatible with the promotion of angiogenesis [34]. On the contrary, NPY, NGF and GAL exhibited their functional role later, towards the proliferative/early remodelling phase.

Differential neuromarker expression was observed at 28 days p.i., especially between calcified and non-calcified tendons, exhibiting temporal and healing-dependent neuromarker patterns. In detail, the expression of NPY, NGF, CGRP and GAL became prevalent in non-calcified tissues 28 days p.i., while NF200 recovered its levels at similar values to pre-injury. In fact, the early nerve ingrowth observed in mice at 7 days p.i. was followed by a process of outgrowth in the non-calcified samples at 28 days p.i., leading to a favourable healing response [18,36]. The high levels recorded at day 28 p.i. for GAL, NGF, CGRP and NPY could be attributable to additional biological roles. In particular, GAL has an anti-inflammatory effect, suggesting its function in tissue repair through the modulation of immune responses [37,38,39,40]. Similarly, NGF appears to be involved in modulating inflammatory events, especially in the later phases of tendon injury. Likewise, CGRP also has an anti-inflammatory role, along with vasodilation, angiogenic, and matrix-modulating effects through different mechanisms [35,41,42,43]. Indeed, in other tissue healing models, such as skin wound healing, CGRP has been associated with collagen remodelling, favouring the progressive replacement of collagen III with collagen I [44]. A similar influence was documented on human tenocytes, exerted through the activation of MMP-3, which is known to degrade collagen III and promote MMP-9 activity [44,45]. Thus, the upregulation of CGRP expression at 28 days p.i. in non-calcified samples may be indicative of the transition from the proliferative to the early remodelling phases. Furthermore, the upregulation of NPY in non-calcified tendons aligns with the findings that autonomic nerve fibres can induce vasoconstriction [46,47], leading to relative hypoxia, which enhances the tensile strength of the tendon by switching the production of collagen type III to type I [18]. Of note, a differentiative balance between the vasoconstrictive actions of NPY and the vasodilatory actions of CGRP characterised the outcome of tendon healing, suggesting that a balanced presence of these two neuromediators is a favourable condition leading to a good recovery of tissue homeostasis. This balance could be crucial for modulating the oxygen and nutrient transfer to the healing area, thereby controlling fine ECM remodelling.

In contrast, calcified tendons exhibited a different neuromarker profile compared to non-calcified tendons. Specifically, these tendons showed a delay in NF200 upregulation, suggesting a role for this neuromarker in predicting the healing process outcome: basal levels are associated with successful healing, while high levels are found in calcified tissues. The elevated NF200 expression in calcified tendons seems to suggest the persistence of an ongoing nerve ingrowth, linked to altered healing trajectories [19]. At the same time, NGF and NPY showed levels notably higher. Overall, excessive and protracted nerve endings in the tendon proper might suggest pro-inflammatory, nociceptive and hypertrophic tissue responses [48]. In line with the NF200 findings, excessive NGF expression has also been associated with inflammatory conditions and chronic pain states [49]. The dramatic increase in NPY expression observed must also be interpreted in the context of the low CGRP levels in calcified tendons. This highlights the higher ratio between vasodilating and vasoconstricting molecules, which may indicate a potentially responsible imbalance that hinders or complicates the healing process [22]. This deviation from the expected balance of these neuromarkers could negatively influence tissue healing through excessive hypoxia, exacerbating tendon stiffness and potentially impeding the optimal recovery of tendon function and flexibility. Furthermore, the high levels of NPY could be linked to its role in promoting the calcification process [50].

Unlike observations in rat and human tendons [19,21,51], a pivotal finding of this study was also the presence of neuromarkers not only accumulated in the ECM but also within tendon cellular populations such as tenocytes and chondrocyte-like cells. In particular, this study showed the presence of several chondrocyte-like cells in the healing tendons at 28 days p.i. These are cells exhibiting features similar to chondrocytes, such as high positivity for Alcian Blue, round nuclei and pericellular lacunae. However, they expressed TNMD, a typical tenogenic and not chondrogenic marker. No current in vitro/in vivo studies have discussed this evidence; however, the current literature demonstrates that chondrocyte-like cells may arise during tendon differentiation [13,14], through the interplay of regulatory intracellular signalling controlling the commitment of common progenitor cells [52]. These cells also exhibited positivity for the analysed neuromarkers, suggesting a possible contribution of neuromarkers in inducing the metaplastic shift toward chondrogenesis and calcification. Specifically, high NGF levels have been recognised to be inductive of chondrogenic and bone differentiation through the activation of the Indian Hedgehog–parathyroid hormone-related protein signalling axis, a pathway which is vital in the proper development of calcification [53,54]. Previous evidence has also shown the crucial role of NPY in promoting chondrocyte hypertrophy [23]. On the contrary, no current evidence demonstrates any involvement of NF200, CGRP or GAL in chondrogenesis. However, the positivity of chondrocyte-like cells for neuromarkers is believed to be associated with cartilaginous metaplasia and ectopic ossification within healing tendons [55].

The active contribution of tenocytes in neuromarker synthesis was confirmed by a double TNMD staining, a tenocyte-related marker [56], with the neuromarkers. This novel finding challenges conventional assumptions regarding neural interactions with the tendon matrix and cells. Notably, in healthy tendons, TNMD and neuromarkers did not co-localise, indicating that during homeostasis, tenocytes are not engaged in neurobiological mediating mechanisms. However, several tenocytes exhibited intracellular positivity for neuromarkers, by day 7 p.i., underscoring a shift where tenocytes could actively participate in neuromodulation. Previous studies have shown, in vitro or in vivo, the positivity in tenocytes for NF200 [32,57], without demonstrating its specific role, NGF [58], CGRP [20,35] and NPY [59]. In particular, intracellular NGF expression could be linked to the regulation of cell–ECM adhesion proteins, influencing tissue remodelling [60], confirmed in this study by its high expression levels at 28 days p.i. It is known that increased intracellular CGRP expression plays a significant role in tissue repair and homeostasis [35]. Regarding NPY, it has been demonstrated that tenocytes are positive for both the molecule itself and for its Y1 receptor, which may exert possible intracellular effects on signalling pathways, gene expression, neurogenesis and mitochondrial function [20,50,61], probably via autocrine/paracrine signalling. Even though no previous studies specifically show GAL expression in tendon cells, the current study also observed its intracellular expression in tenocytes, especially 28 days p.i., possibly inhibiting inflammatory and nociceptive responses to injury [39,62]. Although further investigations are needed to clarify the molecular mechanisms underlying the neuro-paracrine action in the tendon somatic compartment, the traditional view of tendon cells as passive responders to mechanical stimuli can now be revisited, recognising the role of both recipients and generators of neuromarker signals [21,63] able to influence tendon healing.

## 5. Conclusions

In conclusion, the spatiotemporal analysis of neuromarkers involved in tendon healing during the inflammatory and proliferative/early remodelling phases and in diverse morphological healing outcomes (i.e., non-calcified vs. calcified samples) has been explored for the first time in the ATs of a mouse injury model. These findings present novel insights into the involvement in tendon healing of the several neuromarkers belonging to the sensory and autonomic nervous systems. Of note, the intracellular expression of the analysed neuromarkers within the tenocytes and chondrocyte-like cells could influence ECM deposition and remodelling through a direct dialogue with the nervous system. The profiles of NF200, NGF, NPY, GAL and CGRP recorded before and after injury, during the transition from the inflammatory to the proliferative/early remodelling phases (at 7 and 28 days post-injury, respectively), began to define the expression patterns associated with favourable healing. Notably, the early expression of NF200 and its return to baseline levels by day 28 post-injury appear to be particularly relevant and distinct compared to the calcific healing process. Simultaneously, the differential expression observed in calcified tendons helped identify predictive markers of a poor prognosis. In these tissues, the delayed NF200 upregulation, increased NGF levels and higher NPY/CGRP ratio all indicate an imbalanced proliferative/early remodelling phase, which is likely to result in calcified tendons.

Overall, these results suggest a significant pleiotropic influence of key neuromarkers in tendon healing, highlighting their crucial roles in vasoconstriction and vasodilation, which impact the healing outcome. Specifically, the early and late roles of NF200 are predictive of whether tendon healing will progress favourably or unfavourably, such as in cases of calcification metaplasia. This research underscores the translational value of the mouse AT injury model for understanding mechanisms involved in heterotopic mineralisation in human tendons. Additionally, it provides insights into the neurobiological mechanisms underlying early tendon healing and lays the groundwork for identifying neuromarker expression patterns that could predict tendon healing outcomes.

## Figures and Tables

**Table 1:** Antibodies used for IF analysis, together with the secondary antibody concentration.

Primary Antibody	Primary Antibody Dilution	Secondary Antibody	Secondary Antibody Dilution
CGRP (Chemicon, Lansing, NC, USA, polyclonal AB1971)	1:100	Anti-Rabbit AlexaFluor 488 (AP132C Sigma Aldrich, St. Louis, MO, USA)	1:600
GAL (MyBioSource, San Diego, CA, USA, Polyclonal, MBS565327)	1:400	Anti-Rabbit AlexaFluor 488 (AP132C Sigma Aldrich, St. Louis, MO, USA)	1:600
NGF (Sigma Aldrich, St. Louis, MO, USA, Polyclonal, N6655)	1:400	Anti-Rabbit AlexaFluor 488 (AP132C Sigma Aldrich, St. Louis, MO, USA)	1:600
NF200 (Sigma Aldrich, St. Louis, MO, USA, Polyclonal, N4142)	1:400	Anti-Rabbit AlexaFluor 488 (Abcam, Cambridge, UK, Code:AB 150077)	1:600
NPY (Sigma Aldrich, St. Louis, MO, Polyclonal, N9528)	1:500	Anti-Rabbit AlexaFluor 488 (AP132C Sigma Aldrich, St. Louis, MO, USA)	1:600
Tenomodulin (Abcam, Cambridge, UK, ab203676)	1:500	Anti-Rabbit CY3 (Abcam, Cambridge, UK, AB 150077)	1:600

## Data Availability

The data supporting the reported results are available upon request to the authors.
